# Prognostic correlation of *NOTCH1* and *SF3B1* mutations with chromosomal abnormalities in chronic lymphocytic leukemia patients

**DOI:** 10.1002/cnr2.1757

**Published:** 2022-11-21

**Authors:** Reza Sadria, Nasrin Motamed, Mohammad Saberi Anvar, Hassan Mehrabani Yeganeh, Behzad Poopak

**Affiliations:** ^1^ Department of Cell & Molecular Biology, School of Biology, College of Science University of Tehran Tehran Iran; ^2^ Department of Medical Genetics Payvand Clinical and Specialty Laboratory Tehran Iran; ^3^ Department of Animal Sciences, College of Agriculture and Natural Resources University of Tehran Tehran Iran; ^4^ Tehran Medical Sciences Branch Islamic Azad University Tehran Iran

**Keywords:** chromosomal abnormality, chronic lymphocytic leukemia, CLL FISH panel, *NOTCH1*, *SF3B1*

## Abstract

**Background and Aim:**

Chronic lymphocytic leukemia (CLL) is a monoclonal malignancy of B lymphocytes. Since common mutations in *NOTCH1* and *SF3B1*, along with other possible chromosomal alterations, change disease severity and survival of patients with CLL, we aimed to evaluate the correlation of common mutations in *NOTCH1* and *SF3B1* as the poor prognostic markers with chromosomal abnormalities and clinical hematology.

**Method:**

This retrospective study was performed on the peripheral blood of 51 patients diagnosed before chemotherapy with CLL. G‐banding karyotype and FISH were performed. For *NOTCH1*, exon 34 and for *SF3B1*, exons 14,15,16 were assessed using Sanger sequencing.

**Results:**

The mutation frequency of *NOTCH1* and *SF3B1* with the pathogenic clinical status was 6:51 (11.76%), and variants obtained from both genes were 9:51 (17.64%). The frequency of *SF3B1* mutation (K666E) was higher than in previous studies (*p*‐value <.05). There was a significant correlation between *NOTCH1* mutations and del17p13 (*p*‐value = .068), also *SF3B1* mutations with del11q22 (*p*‐value = .095) and del13q14 (*p*‐value = .066). Up to 90% of the specific stimuli used for the G‐banding karyotype successfully identified the malignant clone. There was a significant relationship between the cluster of differentiation 38 (CD38) expression level and *NOTCH1* mutations (*p*‐value = .019) and a significant correlation between Binet classification and the *SF3B1* (*p*‐value = .096).

**Conclusion:**

The correlation of *NOTCH1* and *SF3B1* mutations with chromosomal abnormalities and CD38 expression may reveal the overall patient's survival rate. The mutations may be effective in the clonal expansion and progression of CLL, particularly in the diagnosis stage, as well as the control and management of the treatment.

## 1 INTRODUCTION

Leukemia is one of the most common types of cancer. According to the World Health Organization (WHO), chronic lymphocytic leukemia (CLL) is a monoclonal malignancy of small and mature neoplastic B lymphocytes (CD5^+^) that is associated with CD19^+^ and CD23^+^ expression.^1^, ^2^, ^3^ The CLL is characterized by the presence of ≥5000/μl monoclonal B lymphocytes (single lineage) in peripheral blood for more than 3 months.^3^ Moreover, CLL has an incidence of about 4–5 per 100 000 people and is less reported in Asia and Africa than in Europe and North America, with an average age of about 70 years. The chance of malignancy in men is approximately two times more than in women, and roughly 85% of cases may survive up to 5 years.^4^ About 10% of the patients have a family history related to this leukemia.^3^ Several patients are asymptomatic at the diagnosis. Some patients are stable, even for the rest of their lives, and do not need treatment.^5^

There is a widespread presence of this leukemia in the blood, bone marrow, other lymphoid tissues, and spleen.^6^ Small, mature lymphocytes with a narrow rim of cytoplasm and a dense nucleus without detectable nucleoli and smudge and basket cells can be seen in the blood smear of patients with CLL. A small percentage of large, atypical, proliferative, and small lymphocytes may be seen in atypical CLL (aCLL). The presence of more than 10% prolymphocyte in the CLL may indicate a more invasive form of the disease associated with mutations in the *TP53* and *NOTCH1*.^2^, ^7^ About 99% of tumor cells are in the G0, or early G1 stages of the cell cycle.^8^ Deficiency in apoptosis leads to the accumulation of tumor cells and disease progression. The expression of ZAP‐70, CD49d, and CD38 has a crucial role in the prognosis of the progressive form of CLL and demonstrates poor prognosis.^9^, ^10^

There are several causes for CLL, including genetic and cytogenetic disorders. Approximately 80% of patients with CLL carry at least one of the five most common chromosomal abnormalities (del(6q), del11q23, del13q14.3, del17p13, and Trisomy12).^11^ The low proliferation of B cell clones disrupted the chromosomal analysis.^12^ Standard cytogenetic methods have observed up to 30% of CLL clonal cytogenetic abnormalities, and up to 80% of them have been observed by the Fluorescent In‐situ Hybridization (FISH) technique using specific probes.^13^ Although the standard CLL FISH panel is highly specialized in the prognosis of CLL, shreds of evidence have shown that chromosomal analysis may provide further information.^12^

In the G‐banding karyotype method, using specific stimuli, it is possible to increase the resolution and preserve malignant clones that can be increased in identifying the chromosomal abnormalities, especially Complex Karyotypes (CK). The method provides important information for prognosis of patients with CLL and level classification of disease severity at a lower cost.^12^ Moreover, 32%–42% of the patients with CLL had chromosomal translocations determined by the conventional G‐band karyotype method. The importance of predicting such abnormalities related to chromosomal translocations has been debated. Initially, it was assumed that the presence of translocations was associated with poor clinical outcomes depending on the number of rearrangements, but recent studies have limited the clinical outcomes to translocations observed in the form of complex or unbalanced translocations.^14^

The somatic mutations detected in patients with CLL are classified into two groups based on the status of the immunoglobulin heavy chain gene (*IGHV*) mutations. Recently, mutations reported for diagnosis of CLL in *TP53*, *SF3B1*, *NOTCH1*, and *BIRC3* have indicated poor prognosis and disease progression.^15^, ^16^ *NOTCH1* and *SF3B1* mutations are the most critical mutations in patients with CLL. Information from cytogenetic alterations indicates that if mutations in the mentioned genes are accompanied (del13q, +12), the classification of the level of disease severity changes from low‐risk to high‐risk. These changes reduce patients' survival by ~20%.^17^ *NOTCH1* mutations in CLL occur mainly at exon 34 and often at the end of the PEST domain (a region rich in proline (P), glutamic acid (E), serine (S), and threonine (T)). *NOTCH1* mutations are often reported with unmutated‐*IGHV* and Trisomy 12 and rarely in cases with del13q. In CLL, the missense mutation of the *SF3B1* corresponds to a specific cluster of 3–5 HEAT domain repeats (Huntington, elongation factor 3, PR65/A, TOR). This mutation is more common in cases with normal karyotypes or del11q.^15^

Because the prognosis of Iranian CLL patients is solely based on cytogenetic changes, the presence of common somatic mutations causes clonal expansion and changes the prognostic category. Therefore, the present study aimed to evaluate the prognosis of patients with CLL in the Iranian population by examining the common mutations in *NOTCH1* and *SF3B1* as poor prognostic markers and their correlation with chromosomal abnormalities and clinical hematology.

## 2 MATERIALS AND METHODS

### 2.1 Study design

This retrospective cohort study was performed on patients referred for diagnosis of malignancy before chemotherapy from 2018 to 2020 in the Payvand Clinical and Specialty Laboratory. According to the hematological and immunophenotypical results based on the International Workshop on Chronic Lymphocytic Leukemia (IWCLL) 2018, 82 patients were diagnosed with CLL. Out of 82 patients, 51 patients (ranges 28–88 years) were enrolled in the study because they had molecular cytogenetic results. Demographic and hematologic information and clinical medical history of all participants were recorded and analyzed to determine the Binet stage. Also, surface expression of prognostic markers such as CD38, CD49d, and ZAP‐70 was evaluated by the Flow cytometry method. G‐banding karyotype and I‐FISH (Interphase‐FISH) techniques were used to evaluate chromosomal abnormalities, and sequencing of *NOTCH1* and *SF3B1* was performed by the Sanger method. This study was approved by the Ethics Committee of Tehran University of Medical Sciences (Ethics code: IR.UT.SCIENCE.REC.1401.003). The samples were collected after explaining the protocol to the subjects and obtaining written informed consent from patients. The flowchart of the study is depicted in Supplementary Figure 1.

**FIGURE 1:**
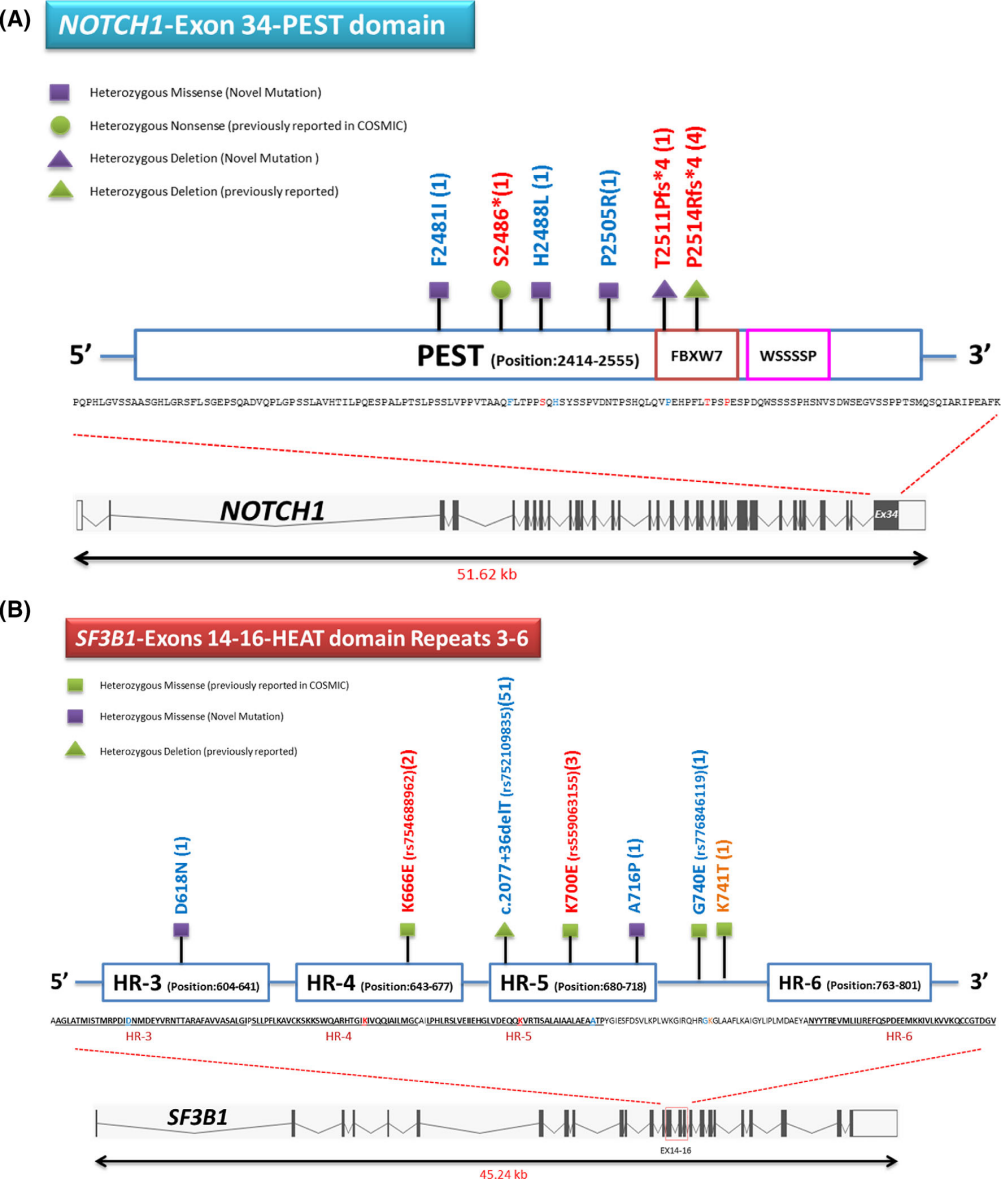
(A) Schematic of the PEST domain and identified mutations in *NOTCH1* gene. (B) Schematic HEAT domain repeats 3–6 and identified mutations in the *SF3B1* gene

### 2.2 Chromosome banding analysis and FISH

A conventional cytogenetic study was performed on the peripheral blood of 20 patients. For the metaphase study, 20 × 10^6^ peripheral blood cells were incubated for 72 h at 37 °C and 5% CO_2_ pressure and were cultured in a T25 flask with a volume of 10 ml of RPMI‐1640 + 25 mM GlutaMAX (Gibco, Invitrogen) medium with 20% Fetal Bovine Serum (FBS, Gibco, Invitrogen), 1% penicillin–streptomycin (10^3^ U/ml) (Gibco, Invitrogen), and 1% MEM non‐essential amino acids solution (Sigma, Aldrich). To stimulate malignant B cell clones in cell culture, 20 μM CpG‐oligonucleotides DSP30 (5′‐TCGTCGCTGTCTCCGCTTCTTCTTGCC‐3′), 2 ml supernatant of Jurkat cell line, which could produce interleukin‐2 (IL‐2) and 100 μl Phytohemagglutinin (PHA) (Gibco) were used. The method used for chromosomal preparation of CLL malignancy was that of Koczkodaj et al.^18^, ^19^ This method was optimized in our laboratory. From each culture, 20 metaphases were studied by G‐banding karyotype. GenASIs BandView version 7.2.7 software (ASI's karyotyping, California, USA) was used to report the karyotype interpretation, according to International System for Human Cytogenetics Nomenclature (ISCN)‐2020. Three or more chromosomal abnormalities in an identical clone were considered complex karyotypes. Clonal abnormalities were those in which structural rearrangement or chromosomal gain was seen in at least two metaphases, and loss of a single chromosome was seen in at least three metaphases.^20^ Also, the status of the studied metaphases was scored based on three parameters: 1‐Metaphase proliferation, 2‐Binding quality, and 3‐Stimulus effect on a malignant clone (Supplementary Table 1).^21^

**TABLE 1:** Relationship of clinical results and NOTCH1 and SF3B1 mutations

				*NOTCH1*					*SF3B1*				
		Total patients		Mutated		Wild‐type			Mutated		Wild‐type		
		No	%	No	%	No	%	Sig‐No	No	%	No	%	Sig‐No
Subject	Number of patients	51	100	8	16	43	84		9	18	42	82	
Sex	Male	33	65	4	12	29	88	0.43	6	18	27	82	1
	Female	18	35	4	22	14	78		3	17	15	83	
Age	>60 year	24	47	5	21	19	79	0.451	4	17	20	83	1
	<60 year	27	53	3	11	24	89		5	19	22	81	
Swelling lymph node	>3 nodes	20	39	4	20	16	80	0.696	5	25	15	75	0.289
	<3 nodes	31	61	4	13	27	87		4	13	27	87	
WBC count	>100 (10^3^/μl)	7	14	1	14	6	86	1	2	29	5	71	0.592
	<100 (10^3^/μl)	44	86	7	16	37	84		7	16	37	84	
Lymphocyte count	>5 (10^3^/μl)	48	94	8	17	40	83	1	8	17	40	83	0.449
	<5 (10^3^/μl)	3	6	0	0	3	100		1	33	2	67	
Hb	>11 (g/dL)	43	84	6	14	37	86	0.595	9	21	34	79	0.322
	<11 (g/dL)	8	16	2	25	6	75		0	0	8	100	
Hct	>33%	43	84	6	14	37	86	0.595	9	21	34	79	0.322
	<33%	8	16	2	25	6	75		0	0	8	100	
Plt count	>100 (10^3^/μl)	42	82	6	14	36	86	0.619	8	19	34	81	1
	<100 (10^3^/μl)	9	18	2	22	7	78		1	11	8	89	
Smudge and basket cell	Present	40	78	6	15	34	85	1	6	15	34	85	0.385
	Absent	11	22	2	18	9	82		3	27	8	73	
Smudge and basket cells grade	Zero	11	22	2	18	9	82	0.833	3	27	8	73	0.705
	Few	18	35	2	11	16	89		2	11	16	89	
	Few to moderate	2	4	0	0	2	100		0	0	2	100	
	Moderate	17	33	3	18	14	82		3	18	14	82	
	Many	3	6	1	33	2	67		1	33	2	67	
Pro‐lymphocyte	Present	15	29	3	20	12	80	0.679	5	33	10	67	0.102·
	Absent	36	71	5	14	31	86		4	11	32	89	
Staging binet	A	33	65	4	12	29	88	0.142^·^	4	12	29	88	0.096·
	B	14	27	2	14	12	86		5	36	9	64	
	C	4	8	2	50	2	50		0	0	4	100	
Immunophenotype	CLL	32	64	0	0	32	100	<0.0001··	5	16	27	84	1
	aCLL	18	36	8	44	10	56		3	17	15	83	
CD38 expression	Positive	13	25	5	38	8	62	0.019··	3	23	10	77	0.676
	Negative	38	75	3	8	35	92		6	16	32	84	

Abbreviations: TS(**·**), tendency to significant; S(**··**), significant.

I‐FISH analysis was performed on the peripheral blood of all patients. The following FISH locus‐specific probes were used for the Standard CLL FISH panel. LSI D13S319 (13q14.3)/LSI 13q34/CEP 12 Probe (MetaSystems, Germany). LSI p53 (17p13.1)/LSI ATM (11q22.3) Probe (MetaSystems, Germany).

The test method was performed according to the Association of Genetic Technologists (AGT) cytogenetics laboratory manual.^22^ Two hundred interphase cells were analyzed with ISIS software version 5.8 (Meta‐system, Germany) for each probe. Those results were considered abnormal if the pattern of abnormal hybridization of the probe was higher than the cut‐off range of the control. Cut‐off value in our laboratory for each of the probes, respectively determined as +12 > 15%, del^17^ (p13) ≥ 10%, del^11^ (q23) ≥ 10%, del^13^ (q14) and del^13^ (q34) > 10%. According to the National Comprehensive Cancer Network (NCCN®) instruction, the risk of cytogenetic changes by the FISH technique was classified into three levels, which are 1—Favorable, 2—Neutral, and 3—Unfavorable^23^; each level corresponds to a series of specific chromosomal changes shown in (Supplementary Table 2).

**TABLE 2:** The relationship of quantification (mean ± SEM) of NOTCH1 and SF3B1 mutations with disease profile status

Parameters	Statistics	Molecular study			
		NOTCH1 Ex34		SF3B1 Ex14‐16	
		Mutation	Wild type	Mutation	Wild type
Age (Year)	Mean	62.25	57.83	55.11	59.26
	SEM	5.19	2.24	4.89	2.26
	*p*‐value	.439		.445	
WBC count (×10^3^/μl)	Mean	37.87	19.7	31	20.74
	Mean (LR)	3.63	2.98	3.43	3.03
	SEM	0.32	0.19	0.34	0.19
	*p*‐value	.109·		.332	
Lymphocyte count (×10^3^/μl)	Mean	53.17	55.58	68.67	52.31
	Mean (LR)	3.97	4.01	4.22	3.95
	SEM	0.46	0.19	0.38	0.2
	*p*‐value	.930		.546	
Hb level (g/dL)	Mean	12.48	13.61	13.27	13.46
	Mean (LR)	2.52	2.61	2.58	2.6
	SEM	0.07	0.02	0.06	0.03
	*p*‐value	.261		.847	
Hct (%)	Mean	37.55	40.7	39.77	40.28
	SEM	2.66	1.17	2.54	1.2
	*p*‐value	.284		.859	
Plt count (×10^3^/μl)	Mean	152	170.85	189.77	162.82
	Mean (LR)	5.02	5.14	5.24	5.09
	SEM	0.16	0.06	0.14	0.07
	*p*‐value	.517		.345	
Cell with CLL phenotype, %CD38	Mean	62.25	57.83	55.11	59.26
	SEM	5.19	2.24	4.89	2.26
	*p*‐value	.439		.445	
Cell with aCLL phenotype, %CD38 ≥ 30%	Mean	57.6	74.87	81	64.4
	SEM	6.58	5.2	8.98	4.93
	*p*‐value	.064 ·		.133 ·	

Abbreviations: LR, logistic regression; SEM, standard error of the mean; TS(**·**), tendency to significant.

### 2.3 Molecular study

#### 2.3.1 Genotyping

Peripheral blood samples were taken from patients with EDTA anticoagulant for somatic DNA extraction. After isolation of mononuclear cells (containing lymphocytes) by Lymphodex buffer (Inno‐train, Kronberg, Germany) using Add prep genomic DNA extraction kit (Add Bio, Daejeon, South Korea), DNA was extracted. The quality of the extracted DNA was measured with BioPhotometer plus 6132 (Eppendorf, Hamburg, Germany). Absorption values of 260/280 between 1.7 and 2 were confirmed.

#### 2.3.2 *NOTCH1* and *SF3B1* Genotyping

For *NOTCH1* (Ref seq: NM_017617.5), exon 34 (PEST domain, amino acid 2414–2555) and for *SF3B1* (Ref seq: NM_012433.4), exons 14, 15, and 16 (HEAT domain repeats 3–6, amino acid 603–790) were selected. These areas were selected due to the high incidence of mutations in patients with CLL.^24^ Polymerase Chain Reaction (PCR) was utilized to amplify the desired fragment. Direct Sanger sequencing was performed using Add start Taq master on 100 ng Genomic DNA (gDNA) with the desired primers with the conditions of performing PCR by PCR‐Thermo cycler (Eppendorf‐LifeIII, Hamburg, Germany), according to the programs summarized in Supplementary Table 3 for both genes. For observing the PCR product, 2% agarose gel with DNA Green Viewer (Parstous Biotechnology, Mashhad, Iran) was used with the UVITEC Gel Documentation systems. The PCR product was purified from agarose gel with GEL/ PCR Purification Kit (Favorgen, Pingtung, Taiwan) and read by bi‐direct Sanger sequencing by ABI‐3500 with eight capillaries and pop7 polymer. The CodonCode Aligner Version 9.0.1 software was used to analyze sequencing chromatograms. Nucleotide changes observed in sequencing were examined in clinical and cancer databases such as Clinvar, Human Gene Mutation Database (HGMD), Franklin, Varsome, Catalogue Of Somatic Mutations In Cancer (COSMIC), Clinical Interpretation of Variants in Cancer (CIViC), International Cancer Genome Consortium (ICGC), GDC Data Portal‐National Cancer Institute (GDC), and cBio Cancer Genomics Portal and Genome Aggregation Database (gnomAD).

#### 2.3.3 Statistical analysis

Statistical analysis of the data was performed using Excel‐Microsoft Office 2010 as well as SPSS‐Version 20. For all quantitative data, frequency, percentage and mean ± standard error of the mean (SEM) were presented with amplitude. Chi‐square (χ^2^) and Fisher exact tests were used for qualitative variables and independent t‐tests for quantitative variables, respectively. A logistic regression procedure was used for the normal distribution of variables for those data counted per unit area. A binomial test was also used to compare the frequency of mutations. Meanwhile, the *p*‐value ≤.05 was considered statistically significant, and 0.05–0.15 was considered a significant tendency.^25^, ^26^

## 3 RESULTS

### 3.1 Clinical data

Fifty‐one CLL patients (33 males (65%) and 18 females (35%)) were evaluated in the diagnosis phase before chemotherapy. The mean age of patients was 58.5 ± 2 years in the range of 28–88 years, with a median age of 60 years. The highest frequency distribution of the disease was related to the age range of 51–70. The male:female ratio was almost double. In the clinical evaluation of the risk of disease based on the Binet classification system, it was found that 33 patients (64.71%) were level A, 14 patients (27.45%) were level B, and four patients (7.84%) were level C. In addition, the classification of genetic alterations and evaluation of immunophenotypic poor prognostic markers with three levels of A/B/C ranking revealed that 15 patients (29.41%), nine patients (17.65%), and 27 patients (52.94%) were at A, B, and C levels, respectively. In determining the immunophenotype of 51 patients, based on IWCLL, 50 patients (98%) were diagnosed with CLL clones and one patient (2%) with Prolymphocytic Leukemia (PLL). Based on prognostic markers (CD38, CD49d, ZAP‐70), 63% showed CLL immunophenotype, and 35% showed aCLL immunophenotype. The expression levels of CD38, CD49d, and ZAP‐70 on the CLL clone positive were 13:51(25.49%), 9:27(33.33%), and 1:12(8.33%) patients, respectively. The relationship between clinical results and *NOTCH1* and *SF3B1* mutations is summarized in Table 1.

An independent sample *t*‐test was used to examine the means of quantitative variables. Parameters such as age, the absolute count of hematological changes, including (WBC, Lymphocyte, Hb, Hct, Plt), CD38 expression in CLL phenotype cells and mean CD38^+^ expression (≥30%) in cells with aCLL phenotype were examined by mutations related to the *NOTCH1*, exon 34, PEST domain and the *SF3B1*, exons 14–16, HEAT domain repeats 3–6 (Table 2).

The cytogenetic results obtained from I‐FISH study with the standard CLL FISH panel are shown in Supplementary Figure 2. According to the NCCN guideline, 13 people (26%) were at a favorable level, 24 people (47%) were at a neutral level, and 14 people (27%) were at an unfavorable level. Also, the results obtained from 20 patients studied by G‐banding karyotype, which used the specific stimulus of B cell clones, could be up to 90% successful based on the three defined goals. This evaluation showed that six patients (30%) had normal karyotype status (without structural changes and number), 10 patients (50%) had less than three chromosomal changes in one clone, which was a favorite, and four patients (20%) had more than three chromosomal changes in a clone, which was one of the unfavorable cases (Table 3).

**TABLE 3:** Results obtained from conventional cytogenetics using the specific stimuli of B cell clones and its relationship with the prognostic status of disease based on FISH assessment and determination of disease immunophenotype

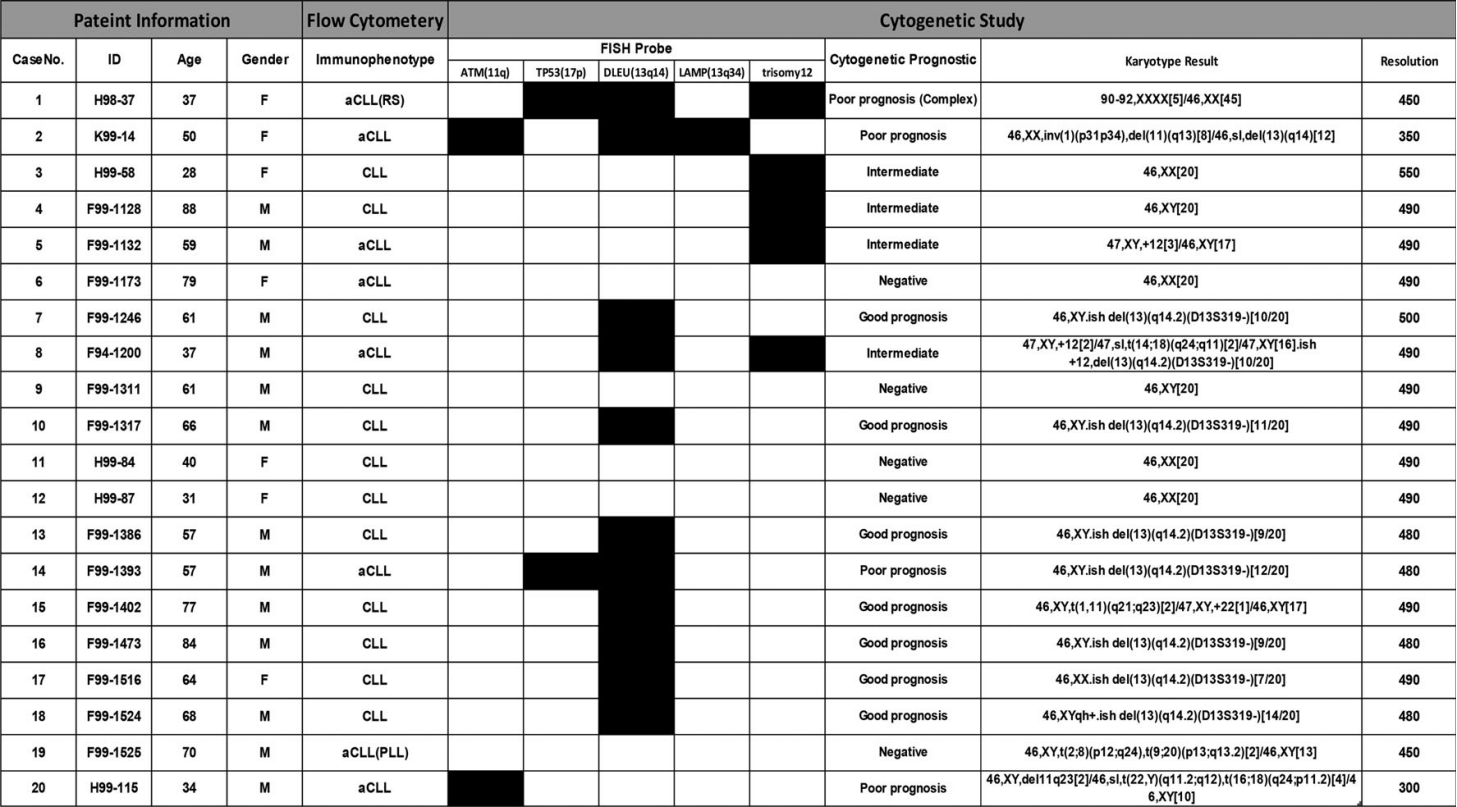

*Note*: Boxes colored black and white show the presence and absence of each item for the patients, respectively.

Abbreviations: aCLL: atypical chronic lymphocytic leukemia; CLL, chronic lymphocytic leukemia; F, female; M, male.

In the analysis of the mutation related to *NOTCH1*, exon 34, PEST domain and *SF3B1*, exons 14–16, HEAT domain repeats 3–6; it was found that out of 51 patients, 32 patients (62.74%) with CLL phenotype, 18 patients (35.29%) with aCLL phenotype, and one patient (1.96%) with PLL phenotype were evaluated. Analysis of the *NOTCH1* showed that nine variants occurred only in eight aCLL patients. One female had two simultaneous mutations (S2486* mutation and P2505R mutation). According to the data, there were three mutations in patients with aCLL, five mutations in those with CLL, and one mutation in those with PLL in the *SF3B1*. The *SF3B1*mutations were observed in nine patients. However, one variant in the deep intronic region of the *SF3B1* between exons 14 and 15 (c.2077 + 36delT) was observed in 51 cases (100%). Also, two simultaneous mutations occurred in one patient, one on the *NOTCH1* (H2488L) and the other on the *SF3B1* (K700E). Figure 1 shows the mutations identified on the PEST domain in the *NOTCH1* and HEAT domain repeats 3–6 in the *SF3B1*. Supplementary Table 4 shows Sanger sequencing description related to gender, variant type, and clinical type status of mutations observed in 51 patients for *NOTCH1* and *SF3B1*.

Six pathogenic mutations (11.76%) and three variations of unknown significance (VUS) mutations (5.78%) were discovered among the mutations examined for *NOTCH1*. In the coding and non‐coding regions, the *SF3B1*, exon 14, and one polymorphism variation as VUS were also found. The remaining variants for *SF3B1* exons 14–16 comprised three VUS mutations (5.88%), one likely pathogenic mutation (1.96%), and five pathogenic mutations (9.80%). Supplementary Table 5 shows clinical results from clinical and cancer databases related to mutations identified for *NOTCH1* and *SF3B1*.

### 3.2 Frequency of *NOTCH1* and *SF3B1* variants and relevance to global studies

Statistical analysis obtained by comparing the frequency of variants in the present study with other global studies recorded in databases (ICGC, NCI‐60, Genom AD, GDC) showed that the frequency of P2514Rfs*4 mutations with 4:51 (7.84%) of *NOTCH1*, despite matching, was not significantly different. However, the variants obtained from *SF3B1* were higher than the frequency of the world studies. There was a significant difference between the frequency of K666E mutation with 2:51 (3.92%) in our study compared to other studies. There was a global record in the ICGC (3:510 (0.58%)) (*p*‐value = .036) and GDC (2:924 (0.22%)) (*p*‐value = .003) databases. The frequency of K700E mutation with 3:51 (5.88%) between our study and the obtained studies from GDC (17:924 (1.84%)) had a significant tendency (*p*‐value = 0.061). A less significant tendency (*p*‐value = .102) was obtained from the ICGC (1:510 (0.20%)) on the G740E mutation with our study 1:51 (1.96%). On the c.2077 + 36delT variation (rs752109835), which was associated with polymorphism, our investigation with 51:51 (100%) and studies collected from the Genome AD (12 510:83948 (14.90%) database found a significant difference (*p*‐value = .0001). In addition, there was a significant difference between the frequency of rs763016003 and rs559063155 mutations in the present study and studies obtained from the Genome AD database (*NOTCH1*: 5:240990 = 0.002% and *SF3B1*: 22:245240 = 0.01%). Supplementary Table 6 shows the significance status of the identified variants in our study compared with global studies achieved from cancer and clinical databases.

### 3.3 Correlation of *NOTCH1* and *SF3B1* mutations with disease‐related clinical components

The statistical analysis of *NOTCH1* mutations showed that there was a significant difference (*p*‐value < .0001) between CLL and aCLL phenotypes. Also, a significant difference (*p*‐value = 0.019) was observed between *NOTCH1* mutation and CD38 expression level. In addition, a significant difference (*p*‐value = .015) was observed by classifying genetic changes and evaluating immunophenotypic poor prognostic markers at three levels of A/B/C. On the other hand, there was a significant tendency for the differences such as del17p13 (*TP53*) (*p*‐value = .068) and Binet classification (*p*‐value = .142).

In another statistical analysis of the *SF3B1* mutations, a significant difference (*p*‐value = .008) was observed in the classification of the mentioned genetic changes and immunophenotypes. Also, there was a significant tendency between *SF3B1* with variables such as proliferation number (*p*‐value = .102), del11q23(*ATM*) (*p*‐value = .095), del13q14 (*DLEU*) (*p*‐value = .066) and Binet classification (*p*‐value = .096). Table 4 shows the correlation results of *NOTCH1* and *SF3B1* mutations with genomic variation components to assess the prognostic status of the disease. Supplementary Figure 3 is an overview of all clinical components and diagnostic and prognostic evaluation of the disease.

**TABLE 4:** Correlation of *NOTCH1* and *SF3B1* mutations with genomic change components to assess the prognostic status of the disease

				*NOTCH1*					*SF3B1*				
		Total patients		Mutated		Wildtype			Mutated		*Wildtype*		
		No	%	No	%	No	%	Sig‐No	No	%	No	%	Sig‐No
Deletion11q22	Positive	7	14	1	14	6	86	1	3	43	4	57	0.095·
	Negative	44	86	7	16	37	84		6	14	38	86	
Trisomy12	Positive	28	55	5	18	23	82	0.715	6	21	22	79	0.487
	Negative	23	45	3	13	20	87		3	13	20	87	
Deletion13q14,q34	Positive	27	53	5	19	22	81	0.707	2	7	25	93	0.066·
	Negative	24	47	3	13	21	88		7	29	17	71	
Deletion17p13	Positive	7	14	3	43	4	57	0.068·	0	0	7	100	0.328
	Negative	44	86	5	11	39	89		9	20	35	80	
Dual changes	Positive	13	25	3	23	10	77	0.404	4	31	9	69	0.208
	Negative	38	75	5	13	33	87		5	13	33	87	
Complex	Positive	5	10	2	40	3	60	0.17	0	0	5	100	0.571
	Negative	46	90	6	13	40	87		9	20	37	80	
Karyotype abnormality	Present	14	70	1	7	13	93	0.521	2	14	12	86	0.549
	Absent	6	30	1	17	5	83		2	33	4	67	
Stimulation efficiency grade	2	6	30	1	17	5	83	0.3	2	33	4	67	0.508
	3	10	50	0	0	10	100		1	10	9	90	
	4	4	20	1	25	3	75		1	25	3	75	
Proliferation grade	3	2	10	0	0	2	100	1	1	50	1	50	0.368
	4	18	90	2	11	16	89		3	17	15	83	
Banding grade	3	2	10	0	0	2	100	1	0	0	2	100	1
	4	18	90	2	11	16	89		4	22	14	78	
Staging genetic and prognostic immunophenotyping marker	A	15	29	0	0	15	100	0.015·	0	0	15	100	0.008··
	B	9	18	0	0	9	100		0	0	9	100	
	C	27	53	8	30	19	70		9	33	18	67	

Abbreviations: TS(·), tendency to significant; S(**··**), significant.

## 4 DISCUSSION

The main purpose of this study was to determine the prognostic status of patients with CLL by examining the common mutations in *NOTCH1* and *SF3B1* and their correlation with chromosomal abnormalities and clinical hematology. Background studies have shown that the prognostic status of CLL disease has been reported based on chromosomal changes assessed with the FISH technique. According to previous information, cytogenetic changes by FISH technique cannot be considered an accurate criterion for determining the prognostic status of patients.^17^ Although the FISH technique is a gold standard method with high specificity in the diagnostic stage, the CLL FISH panel is limited to the chromosomal changes in common loci that the probe is designed and cut‐off range: for deletions and rearrangements and fusion between 5% and 10% and numerical abnormalities of about 20%. Also, G‐banding karyotype is cost‐effective and its analysis can provide more information than CLL FISH panel for detection of other possible chromosomal changes.^14^ So far, various solutions have been proposed for the study of genetic disorders and mutations in CLL, including Sanger sequencing, advanced sequencing, whole exome sequencing (WES), and whole genome sequencing (WGS) (detection limit: 10%).^12^, ^16^ Given that CLL is known as a silent disease,^12^ the detection limit of each method may prohibit the accurate identification of genetic disorders, so the relationship between these possible changes can shed light on this vision.

The frequency of *NOTCH1* mutation, known as one of the most common and key mutations in the diagnosis stage of CLL, increases during the disease progression. The *NOTCH1* mutation correlates with disease exacerbation and leads to more treatment problems. The *NOTCH1* mutations have been associated with trisomy 12 in about 40%. One of the most important mutations of the *NOTCH1* in patients with CLL, with approximately 90%, is located in exon 34 of the PEST domain. Approximately 80% of the aforementioned mutation was frameshift deletion with clinical pathogenicity for P2514Rfs*4 at position c.7541–7542 with canonical delCT mutation. Due to the absence of FBXW7 and WSSSSP domains and the loss of lysine residue at the end of the C‐terminal, resistance to protein degradation by the proteasome complex occurs, resulting in *NOTCH1* signaling stability. As a result, increased cell survival and resistance to apoptosis occur. About 10% of the remaining mutations are related to the intracellular domains of the NOTCH1, including TAD (~3%), ANK (~1%), and the 3’‐UTR region (~6%).^15^, ^16^

Maleki et al. showed that the frequency of the mentioned mutation was 10% compared to the control group, and there was a significant difference (*p*‐value = .049) between level III and levels 0‐II for patients with *NOTCH1* mutation for determining the level of disease severity based on Rai classification.^27^ Our study showed that the recorded percentage of frequency for the mutation (rs763016003) is 7.84%. Despite the low number of samples examined from patients with CLL, the evidence indicates that the frequency of our study is consistent with the global studies despite the lack of significance. Also, out of nine variants recorded in our study, four variants were related to P2514Rfs*4, so the frequency is about 45% (4:9), but compared to the pathogenic mutations recorded, this frequency increased to 67% (4:6). The S2486* is a nonsense mutation with the clinical condition of the pathogenic; as a result, this mutation shortens the translated sequence and lacks the FBXW7 and WSSSSP domains, which have been registered (COSM28658) for the *NOTCH1* mutation. Studies in the cancer database showed no evidence for other *NOTCH1* mutations obtained from the present study (Supplementary Table 5) and it is considered a novel mutation. The T2511Pfs*4 on position c.7531–7532 consists of a 2‐bp (AC)‐frameshift deletion with pathological conditions. As a result of an investigation of WES analysis on patients with B cell monoclonal CLL in the mentioned position, another mutation, as T2511Mfs*75 frameshift deletion with the *IGHV*3‐53 mutant, was registered in the Institute of Oncology of the University of Spain (IUOPA‐Instituto de Oncología de Asturias) (http://cbioportal.org/).

The *SF3B1* mutation leads to incorrect splicing in the transcription process, affecting the pathogenicity of CLL, which is a poor prognostic factor for disease survival and development.^28^ Maleki et al. showed a frequency of 12% compared to 1.9% of the control group for the *SF3B1* mutation significantly (*p*‐value = .012).^27^ *SF3B1* mutations have been accumulated in the HEAT domain repeats 3–6 and exons 14–16. Further frequent mutations have been reported in this region, including K700E, G742, K666, and H662, with frequencies of 50%, 19%, 12%, and 4%, respectively.^29^ Our study showed that the recorded frequency for K700E mutation (rs559063155) is 5.88%, which despite the low number of samples examined from patients with CLL, the evidence of a slight increase in the frequency despite no significance compared to global studies. Since out of nine variants recorded in our study, three variants were related to K700E, the frequency was about 33% (3:9), but it increased to 50% compared to the pathogenic mutations (3:6). Regarding K666E mutation, the frequencies obtained in our study are significantly different from the frequencies recorded in ICGC (*p*‐value = .036) and GDC (*p*‐value = .003) databases, while there is no significant difference between the data of these two databases (*p*‐value = .272). In the case of K741T mutation, the frequencies recorded in the ICGC database (0.50%) are not significantly different (*p*‐value = .268) from the data obtained in our study (1.96%). In the present study, D618N mutation was observed for the *SF3B1*, according to Oscier et al.'s study. They demonstrated D618Y mutation with pathogenic status with ID (COSM1159838) in the mentioned amino acid position.^30^

Studies on the structure of the SF3B1 protein indicate that the lysine loci 666, 700, and 742 are probably related to the enzyme's active site.^31^, ^32^ The current study showed that the frequency recorded for the variant (c.2077 + 36delT) was 100% that this variant was observed in all patients studied and there was a significant difference between our study and Genom AD with 14.90% (*p*‐value <.0001). Therefore, the observed frequency of *NOTCH1* and *SF3B1* mutations with clinical pathogenic status of six samples in this study is 11.76% for patients in the diagnosis stage. There is a relative correlation with the frequency obtained from the cancer database for patients with CLL. The total frequency of the present clinical study, including pathogenic and VUS for *NOTCH1* and *SF3B1* with nine variants, was 17.64%, which is slightly higher than the declared frequency worldwide.

There is a unique relationship between *NOTCH1* mutation and *SF3B1*, *BIRC3*, and *MYD88* mutations in patients with CLL. Evidence shows that between 1.2% and 2.6% of patients with CLL have *NOTCH1* and *TP53* mutations simultaneously.^16^ We showed that only one of the studied patients harbored two simultaneous mutations, one in the *SF3B1* (K700E) and the other in the *NOTCH1* (H2488L), with a frequency of 1.96%. One of the reasons that can make this correlation less obvious may be related to the 10%–15% detection limit of the Sanger sequencing method, especially at the diagnosis stage when clonal development is less. Gutierrez et al. indicated recurrent copy number alterations of clones in patients that generally had one of the five most common changes, including deletions on chromosomes 6q, 11q, 13q, 17p and +12, except for deletion of 13q, which is considered a low‐risk class. The remaining changes indicated an increased risk of disease.^33^ An integrated prognostic model that encompasses *NOTCH1*, *SF3B1*, and *BIRC3* mutations along with cytogenetic abnormalities detected by FISH, classifies patients into four distinct prognostic subtypes: high risk (*TP53* and/or *BIRC3* abnormalities), moderate risk [*NOTCH1* and/or *SF3B1* mutations and/or del11q], low‐risk (trisomy 12 and wild‐type for all genetic lesions), and very low‐risk [del(13q) only]. The 10‐year survival rates for the above four subgroups are 29%, 37%, 57%, and 69%, respectively.^23^

The study conducted by Rosati et al. on *NOTCH1* mutation in patients with CLL showed that it was present in both clonal and subclonal states. Accordingly, a *NOTCH1* mutation is a secondary event that occurs from the onset of del11q, Trisomy 12, del17p. The data suggest that *NOTCH1* mutation is not a temporary event but is detected in high‐risk patients.^16^ We showed no significant relationship between *NOTCH1* mutation and the examined chromosomal changes using FISH. However, it is noteworthy that there is a correlation with a significant tendency (*p*‐value = .068) between *NOTCH1* mutation and del17p13 (*TP53*). Out of eight patients (16%) with *NOTCH1* mutation, three patients (38%) were observed with deletion of *TP53*. Correspondingly, there is a significant tendency (*p*‐value = .111) of the clinical status of *NOTCH1* mutation with the deletion of *TP53*. Furthermore, as mentioned in previous studies, since there is a significant relationship between the del17p13 (*TP53*) and its mutation, as well as there is a correlation between the *TP53* mutation and *NOTCH1* mutation, it may be concluded that *TP53* deletion has a correlation with *NOTCH1* mutation. The results of our study were in line with the mentioned study, but the sample size should be increased to approve the significance. The previous study showed that mutations, including *ATM, TP53*, and *SF3B1* were subclonal, occurring as a secondary event in the development of leukemias. About 70% of the *SF3B1* mutations were subclonal, along with del13q and about 30% were associated with del11q. These studies revealed the concept that most *SF3B1* mutations are subclonal, and these results suggest that although *SF3B1* mutations do not initiate CLL, they may later promote disease progression. Studies have shown *ATM, TP53*, and *SF3B1* mutations turn into clonal mutations over time.^29^

We showed that there was no significant relationship between *SF3B1* mutation and any of the examined chromosomal abnormalities, but there was a correlation with a significant tendency (*p*‐value = .095) between *SF3B1* mutation and del11q23 (*ATM*). Of nine patients (18%) with *SF3B1* mutation, three patients (33%) had *ATM* deletion and there is a correlation with a significant tendency (*p*‐value = .066) between *SF3B1* mutation and del13q14 (*DLEU*). Moreover, of nine patients (18%) with *SF3B1* mutations, only two patients (22%) were observed with *DLEU* deletion. We showed a significant correlation (*p*‐value = .045) between del11q23 (*ATM*) and the clinical status of *SF3B1* mutation. It shows a correlation between our results and previous studies. Puiggros et al.'s review study showed a strong association between *NOTCH1* mutation frequency and trisomy 12 in CLL patients with UM‐IGHV or ZAP‐70^+^. Trisomy 12 is mainly used as a clonal driver mutation as an early event in CLL evolution and leads to secondary chromosomal changes and causes mutations in *NOTCH1*, *TP53*, and *FBXW7*.^14^

We showed that there was no significant relationship between trisomy 12 with *NOTCH1* (*p*‐value = .715) and *SF3B1* (*p*‐value = .487) mutations. Moreover, these results indicate that although more than half of the mutations have been recorded along with trisomy12, they do not show significance, unlike the reported literature. There is no clear perception of the etiology of trisomy 12 in patients with CLL. Occurrence of trisomy 12 in the diagnosis stage is a clonal driver in patients with CLL. Resistance to chemotherapy and Richter syndrome develop over time from the onset of the disease. This leads to increased rates of mutations such as *TP53*, *NOTCH1*, *SF3B1*, and *BIRC3*.^17^

Puiggros et al. reported that the G‐banding karyotype was used to explain other chromosomal abnormalities. One of the major limitations of the G‐band karyotype method in patients with CLL is the low rate of tumor metaphase cells. The findings indicate that I‐FISH cannot detect chromosomal abnormalities in the form of complex and heterogeneous. Research has shown that specific B cell stimuli, including CD40 or CpG CpG Oligodeoxynucleotide (ODN) + IL‐2, promote the growth of CLL cells in vitro and increase the detection of chromosomal abnormalities by up to 80%.^14^

The use of DSP30 and IL‐2 as mitotic stimuli has been demonstrated in increasing malignant B cells, especially in CLL. The use of these stimuli has led to an increase in the rate of abnormalities revealed by a cytogenetic study on many cases in comparison to FISH study.^12^ Other advantages of these mitotic stimuli are as follows: first, it increases 98.8% of metaphase appearance, which leads to the identification of 83% of chromosomal abnormalities in patients. Second, it identifies other additional chromosome abnormalities. Identification and diagnosis of complex karyotypes (chromosomal abnormalities ≥3) provide information about the prognostic status of patients. Also, the use of DSP30 and IL‐2 mitotic stimuli at low levels of neoplasia (less than 10%) increases the ability to diagnose abnormalities, while the lipopolysaccharide (LPS) stimulator does not have this ability at a low level.^12^

Wren et al. showed the identification of chromosomal abnormalities in cultures with the presence of 12‐O‐tetradecanoylphorbol 12‐myristate 13‐acetate (TPA) + DSP30 + IL‐2 (52.9%) mitogens in comparison to TPA (45.8%) and DSP30 + IL‐2 (29.2%) mitogens. They found the previous mitogens were not repeatable. On the other hand, no new clonal abnormalities were observed in DSP30 + IL‐2 cultures, unlike TPA. In addition, the success of culture with TPA (100%) versus DSP30 + IL‐2 (72.7%) was that the band resolution observed with TPA was more successful.^13^ Our results indicated that the G‐banding Karyotype was unsuccessful in detecting interstitial deletions with a genome size range <5–10 Mb, especially 13q14 region.^34^ One of the main reasons for not observing was the low resolution of this method. Our findings show that conventional karyotype has not been successful for cases with positive borderline abnormalities seen in FISH technique. These results refute past findings.

Data collected by Navarro et al. showed that trisomy 12 and 13q (13q12‐q32) were the most common chromosomal abnormalities that occurred in patients with CLL, and their incidence is reported to be 12%–32% and 10%–28%, respectively. These two types of abnormalities produce independent single clones for specific CLL subtypes. Simultaneous abnormalities related to +12 and del13q rarely occur in patients with CLL, but the findings in this study showed that there is a correlation between +12 and del13q (RB1, D13S319, and D13S25). The heterogeneous distribution of +12 and del13q occurs heterogeneously in B cell neoplastic cells, suggesting a secondary change in CLL malignancies rather than a primary event.^35^

According to our findings, it is inferred that although a single chromosomal change related to +12 and del13q has the highest frequency percentage, the declared percentage of this study is different from the percentage of the word frequency. About (20%) of the abnormalities in this study are classified as dual and multiple abnormalities that are part of multiple abnormality changes, indicating that patients have a secondary change in distribution a clone occurred.

CD38 and *IGHV* mutation status were prognostic factors in CLL. CD38 expression was different in the progression of CLL. ZAP‐70 expression is associated with *IGHV* mutation status and disease progression.^36^ CD38, CD49, and ZAP‐70 are poor prognostic markers associated with *NOTCH1* mutation.^16^

Puiggros et al. found that atypical immunophenotype occurs in patients with del17p, which increases the severity of CD20, FMC7, and CD79b, as well as the expression levels of CD38, ZAP‐70 and un‐mutant IGHV.^14^ Our findings showed that the level of CD38 expression (cut‐off value >30%) significantly (*p*‐value = .019) correlated with *NOTCH1* mutation and clinical status of *NOTCH1* mutation (*p*‐value = .05), which is consistent with the previous studies. There was also a significant tendency (*p*‐value = .064) in the mean expression of CD38^+^ with a value of 57.6 ± 6.58 in patients with the aCLL phenotype for *NOTCH1* mutation. On the other hand, the expression level of CD38 with parameters such as del11q23 (*ATM*), del17p13 (*TP53*) and multiple abnormalities showed significant tendency (*p*‐value = .09, *p*‐value = .09, and *p*‐value = .087), respectively.

Our study showed that aCLL with parameters such as del17p13, multiple abnormalities (Complex). Also, the effectiveness of the specific mitotic stimuli used in G‐banding karyotype was significant (*p*‐value = .016, *p*‐value = .044, and *p*‐value = .013), respectively.

The Integrated CLL scoring system (ICSS) divided patients into three groups based on cytogenetic abnormalities detected by FISH, *IGHV* mutation status, and CD38 expression to classify risk groups (low, medium, and high). The International Predictive Index for CLL (CLL‐IPI) classifies patients into four risk groups (low, moderate, high, and very high) based on *TP53* and *IGHV* mutation status, serum beta‐2 microglobulin (B2M) concentration, clinical stage, and age. The 5‐year overall survival rates varied significantly between these risk groups (93%, 79%, 63%, and 23%, respectively).^23^ The findings of our study indicate that in many cases, classification based on the set of genetic and prognostic immunophenotyping markers of the disease shows a higher risk than Binet classification. These findings suggest that various factors, including biochemistry, hematology, genetics, and immunophenotype, should be considered in presenting a prognostic model that plays an essential role in determining the fate of patients to estimate the risk of disease with an approximate ratio. The study had some limitations. The mutation states discovered in both genes cannot be linked to a particular race because the patients who were referred to the Payvand Laboratory were related to no specific race. In this study, small size of patients was examined due to the expensive cost of several exams. The number of study participants should be raised in order to achieve a significant difference in the parameters under investigation.

## 5 CONCLUSION

Given the diagnosis phase of patients with CLL, the percentage of common *NOTCH1* and *SF3B1* mutations, which play an important role in the prognosis of patients, was consistent with global studies. There was also a high tendency for correlation between common chromosomal abnormalities of patients with CLL with these mutations, but considering that trisomy 12 was seen as a single or multiple changes in most patients, its effect on the etiology of the disease is still unknown. Furthermore, as a clonal driver in patients with CLL, it has been suggested that it causes secondary disorders and the development and progression of the disease and increases the mutation rate. Although the specific and standard B cell mitogen can be successful in detecting chromosomal abnormalities (structural and numerical), it cannot replace FISH technique due to the limitations of detecting microdeletions and not detecting abnormalities in borderline positive situations. Most disease management approaches partially prevented the disease progression. To prevent the clonal expansion and progression of the disease and guarantee that the therapeutic procedure is successful, it is advised that common mutations that are poor prognostic markers be evaluated along with chromosomal and clinical hematological changes. Finally, one must assert that the issue with this study is the small sample size, which makes definitive conclusions partly uncertain.

## AUTHOR CONTRIBUTIONS

**Reza Sadria:** Conceptualization (equal). **Mohammad Saberi Anvar:** Investigation (equal). **Hassan Mehrabani Yeganeh:** Formal analysis (lead). **Behzad Poopak:** Supervision (lead).

## CONFLICT OF INTEREST

The authors have stated explicitly that there are no conflicts of interest in connection with this article.

## ETHICS STATEMENT

Ethics committee approval codes: IR.UT.SCIENCE.REC.1401.003.

## Supporting information


**Supplementary Figure 1.** Flowchart of the study cohort showing inclusion and exclusion criteria.
**Supplementary Figure 2.** A: Separation of results obtained from I‐FISH with CLL panel probes by sex B: Frequency percentage of each chromosomal change observed in the FISH study.
**Supplementary Figure 3.** Overview of all clinical components, diagnostic and prognostic evaluations related to the disease. Boxes colored black, white, and gray show the presence, absence and no data of each item for the patients, respectively. Coloring pattern is specified for each item based on the variety of changes.

**Supplementary Table 1.** Scoring table
**Supplementary Table 2.** Risk of cytogenetic changes investigated by I‐FISH technique in three levels, 1‐Favorable, 2‐Neutral, and 3‐Unfavorable
**Supplementary Table 3.** Sequence of primers designed for *NOTCH1*, exon 34 and *SF3B1*, exons 14–16 with a schedule for product amplification by PCR
**Supplementary Table 4.** Description of Sanger sequencing related to gender, determination of the variant type and clinical type status of NOTCH1 and SF3B1 mutations in 51 patients
**Supplementary Table 5.** Clinical results from clinical and cancer databases related to mutations identified for NOTCH1 and SF3B1
**Supplementary Table 6.** Significant status of identified variants in the present study compared with global studies achieved from cancer and clinical databases

## Data Availability

The authors declare that all the data of this study will be available.
