# Supplementary material for: Prognostic correlation of NOTCH1 and SF3B1 mutations with chromosomal abnormalities in chronic lymphocytic leukemia patients
Source: Cancer Rep (Hoboken). 2022 Nov 21;6(3):e1757. doi: 10.1002/cnr2.1757 (PMC10026310; doi:10.1002/cnr2.1757)
Supplement: Supplementary file 1 — Supplementary Figure 1. Flowchart of the study cohort showing inclusion and exclusion criteria. Supplementary Figure 2. A: Separation of results obtained from I‐FISH with CLL panel probes by sex B: Frequency percentage of each chromosomal change observed in the FISH study. Supplementary Figure 3. Overview of all clinical components, diagnostic and prognostic evaluations related to the disease. Boxes colored black, white, and gray show the presence, absence and no data of each item for the patients, respectively. Coloring pattern is specified for each item based on the variety of changes. [file CNR2-6-e1757-s002.docx]

**
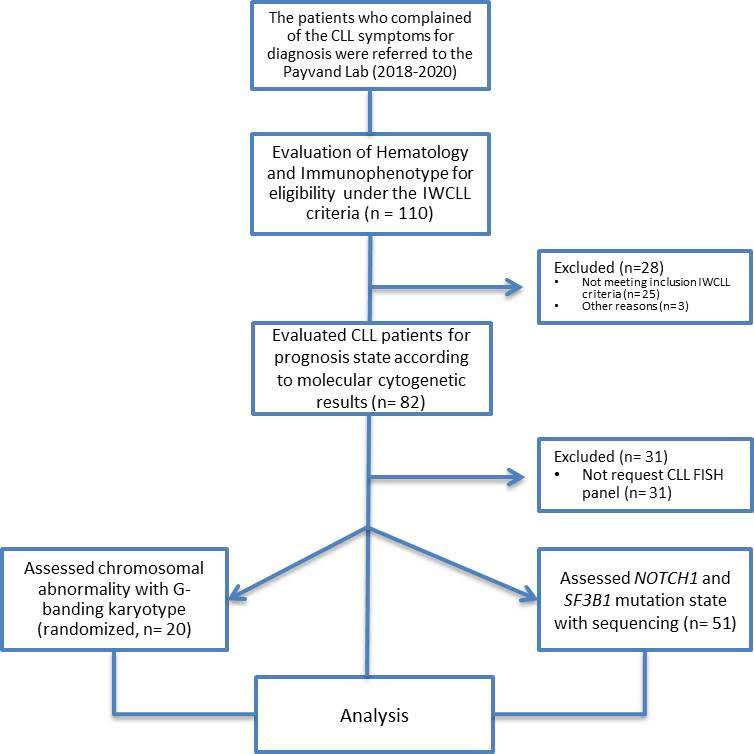
**

**Supplementary Figure 1.** Flowchart of the study cohort showing inclusion and exclusion criteria.


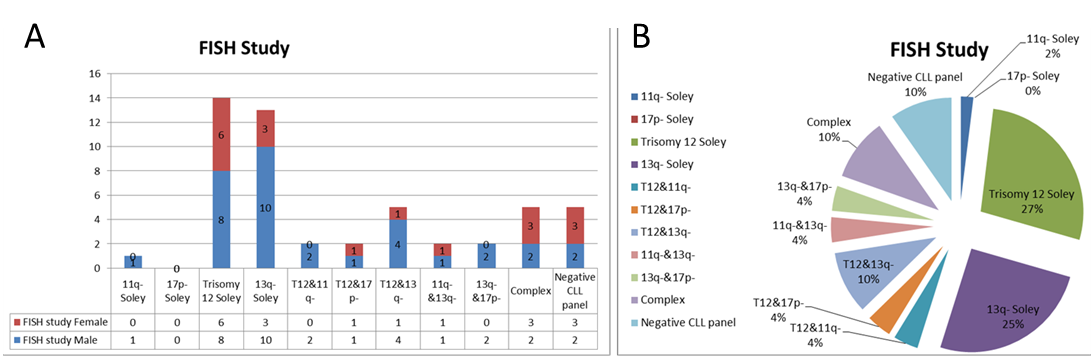


**Supplementary Figure 2.** A: Separation of results obtained from I-FISH with CLL panel probes by sex B: Frequency percentage of each chromosomal change observed in the FISH study.

**Supplementary Figure 3.** Overview of all clinical components, diagnostic and prognostic evaluations related to the disease. Boxes colored black, white, and gray show the presence, absence and no data of each item for the patients, respectively. Coloring pattern is specified for each item based on the variety of changes.
