# Supplementary material for: Prognostic correlation of NOTCH1 and SF3B1 mutations with chromosomal abnormalities in chronic lymphocytic leukemia patients
Source: Cancer Rep (Hoboken). 2022 Nov 21;6(3):e1757. doi: 10.1002/cnr2.1757 (PMC10026310; doi:10.1002/cnr2.1757)
Supplement: Supplementary file 2 — Supplementary Table 1. Scoring table Supplementary Table 2. Risk of cytogenetic changes investigated by I‐FISH technique in three levels, 1‐Favorable, 2‐Neutral, and 3‐Unfavorable Supplementary Table 3. Sequence of primers designed for NOTCH1, exon 34 and SF3B1, exons 14–16 with a schedule for product amplification by PCR Supplementary Table 4. Description of Sanger sequencing related to gender, determination of the variant type and clinical type status of NOTCH1 and SF3B1 mutations in 51 patients Supplementary Table 5. Clinical results from clinical and cancer databases related to mutations identified for NOTCH1 and SF3B1 Supplementary Table 6. Significant status of identified variants in the present study compared with global studies achieved from cancer and clinical databases [file CNR2-6-e1757-s001.docx]

**Supplementary table 1.** Scoring table

| Proliferation |
| --- |
| Score 1: Failure, defined by the presence of 0-1 metaphases |
| Score 2: Poor proliferation, defined by the presence of 2–10 metaphase |
| Score 3: Moderate proliferation, defined by the presence of 11-19 metaphase |
| Score 4: Good proliferation, defined by the presence of ≥20 metaphase |
| Quality of Banding |
| Score 1: Insufficient quality for karyotyping (<200 visible chromosome bands) |
| Score 2: Poor quality (<300 visible chromosome bands) |
| Score 3: Sufficient quality (300–450 visible chromosome bands) |
| Score 4: Good quality (>450 visible chromosome bands) |
| Stimulation Efficiency |
| Score 1: Failure, less than 10 analyzable metaphases |
| Score 2: Normal, absence of clonal abnormalities |
| Score 3: Abnormal I, <3 unrelated chromosome abnormalities |
| Score 4: Abnormal II, >3 unrelated chromosome abnormalities (Complex Karyotype) |

**Supplementary table 2.** Risk of cytogenetic changes investigated by I-FISH technique in three levels, 1-Favorable, 2-Neutral, and 3-Unfavorable

| Interphase cytogenetic (FISH) category | | |
| --- | --- | --- |
| Unfavorable | Neutral | Favorable |
| 11q-, Dual changes (along with 11q-) | Normal | 13q-Soley |
| 17p-, Dual changes (along with 17p-) | +12 |  |
| Complex | +12 and 13q-^(1)^ |  |

1. Van Dyke DL, Shanafelt TD, Call TG, Zent CS, Smoley SA, Rabe KG, et al. A comprehensive evaluation of the prognostic significance of 13q deletions in patients with B-chronic lymphocytic leukaemia. Br J Haematol [Internet]. 2010 Feb 1 [cited 2022 May 24];148(4):544–50.

**Supplementary table 3.** Sequence of primers designed for *NOTCH1*, exon 34 and *SF3B1*, exons 14-16 with a schedule for product amplification by PCR

| Program PCR | Amplicon Size | Sequencing | Primer Name | Gene | Method |
| --- | --- | --- | --- | --- | --- |
| Initial Denaturation at 94 ˚C for 7 min | 537 bp | 5′-AACATCCAGCAGCAGCAAAG-3′ | NOTCH1 Ex34-F | *NOTCH1* | Sanger |
| 35 cycles of 95 ˚C for 40 sec, 64 ˚C for 40 sec*,* 72 ˚C for 40 sec |  | 5′-CATCCACAGAGCGCACAC-3′ | NOTCH1 Ex34-R |  |  |
| Final elongation at 72 ˚C for 7 min |  |  |  |  |  |
| Initial Denaturation at 94 ˚C for 7 min | 496 bp | 5′-GGAATTGATTATGGAAAGAAATGGTTG-3′ | SF3B1 Ex14-F | *SF3B1* |  |
| 35 cycles of 95 ˚C for 40 sec, 63.5 ˚C for 40 sec*,* 72˚ C for 40 sec |  | 5′-GCTGTGTGTGTACCTCTAGTC-3′ | SF3B1 Ex14-R |  |  |
| Final elongation at 72 ˚C for 7 min |  |  |  |  |  |
| Initial Denaturation at 94 ˚C for 7 min | 645 bp | 5′-GAGAGAATCTGGATGATATTGTG-3′ | SF3B1 Ex15/16-F |  |  |
| 35 cycles of 95 ˚C for 40 sec, 61.5 ˚C for 40 sec*,* 72 ˚C for 40 sec |  | 5′-CTGACCTGAAATGAAGAGAATAC-3′ | SF3B1 Ex15/16-R |  |  |
| Final elongation at 72 ˚C for 7 min |  |  |  |  |  |

**Supplementary table 4.** Description of Sanger sequencing related to gender, determination of the variant type and clinical type status of *NOTCH1* and *SF3B1* mutations in 51 patients

| NOTCH1 Ex34 | No | % | SF3B1 Ex14 | No | % | SF3B1 Ex15-16 | No | % |
| --- | --- | --- | --- | --- | --- | --- | --- | --- |
| Male mutant | 4.00 | 7.84% | Male mutant | 2.00 | 3.92% | Male mutant | 4.00 | 7.84% |
| Female Mutant | 4.00 | 7.84% | Female Mutant | 1.00 | 1.96% | Female Mutant | 2.00 | 3.92% |
| Total Patient | 51.00 | 100.00% | Total Patient | 51.00 | 100.00% | Total Patient | 51.00 | 100.00% |
| Coding Impact | No | % | Coding Impact | No | % | Coding Impact | No | % |
| Inframe (Del-Ins) | 0.00 | 0.00% | Inframe (Del-Ins) | 0.00 | 0.00% | Inframe (Del-Ins) | 0.00 | 0.00% |
| Frameshift | 5.00 | 9.80% | Frameshift | 0.00 | 0.00% | Frameshift | 0.00 | 0.00% |
| Missense | 3.00 | 5.88% | Missense | 3.00 | 5.88% | Missense | 6.00 | 11.76% |
| Nonsense | 1.00 | 1.96% | Nonsense | 0.00 | 0.00% | Nonsense | 0.00 | 0.00% |
| Noncoding | 0.00 | 0.00% | Noncoding | 51.00 | 100.00% | Noncoding | 0.00 | 0.00% |
| Clinic Variant | No | % | Clinic Variant | No | % | Clinic Variant | No | % |
| Benign | 0.00 | 0.00% | Benign | 0.00 | 0.00% | Benign | 0.00 | 0.00% |
| VUS | 3.00 | 5.88% | VUS | 1.00 | 1.96% | VUS | 2.00 | 3.92% |
| Pathogenic | 6.00 | 11.76% | Pathogenic | 2.00 | 3.92% | Pathogenic | 3.00 | 5.88% |
|  |  |  |  |  |  | Likely Pathogenic | 1.00 | 1.96% |

**Abbreviation:** Del: Deletion, Ins: Insertion; VUS: variants of uncertain significance

**Supplementary table 5.** Clinical results from clinical and cancer databases related to mutations identified for *NOTCH1* and *SF3B1*

**Supplementary table 6.** Significant status of identified variants in the present study compared with global studies achieved from cancer and clinical databases

| **NOTCH1-Exon 34-PEST domain** | **Our study Vs ICGC** | **Our study Vs GDC** | **Our study Vs G-AD** | **ICGC Vs GDC** | **ICGC Vs G-AD** | **GDC Vs G-AD** |
| --- | --- | --- | --- | --- | --- | --- |
| P2514Rfs*4 | 1 | Not detected | **<0.0001** | Not detected | **<0.0001** | Not detected |
| **SF3B1-Exons14-16-HEAT domain Repeats 3-6** | **Our study Vs ICGC** | **Our study Vs GDC** | **Our study Vs G-AD** | **ICGC Vs GDC** | **ICGC Vs G-AD** | **GDC Vs G-AD** |
| K666E | **0.036** | **0.003** | Not detected | 0.272 | Not detected | Not detected |
| K700E | 0.15 | 0.061 | **<0.0001** | 0.509 | **<0.0001** | **<0.0001** |
| G740E | 0.102 | 0.175 | Not detected | 0.477 | Not detected | Not detected |
| K741T | 0.268 | Not detected | Not detected | Not detected | Not detected | Not detected |
| c.2077+36delT | Not detected | Not detected | **<0.0001** | Not detected | Not detected | Not detected |

**Abbreviations:** ICGC: International Cancer Genome Consortium; GDC: GDC Data Portal-National Cancer Institute; G-AD: Genome Aggregation Database
